# Supplementary material for: Quantifying Land Use/Land Cover and Landscape Pattern Changes and Impacts on Ecosystem Services
Source: Int J Environ Res Public Health. 2019 Dec 23;17(1):126. doi: 10.3390/ijerph17010126 (PMC6981947; doi:10.3390/ijerph17010126)
Supplement: Supplementary file 1 [file ijerph-17-00126-s001.pdf]

Supplementary Material

Quantifying land use / land cover and landscape pattern changes and impacts on ecosystem services

ZHAO Qingjian [*] WEN Zuomin CHEN Shulin DING Sheng ZHANG Minxin

College of Economics and Management, Nanjing Forestry University, Nanjing 210037, China

* Correspondence: zhao5190@126.com

This study is based on the remote sensing images and economic and social development data of Guizhou Province in 2000, 2008, 2013 and 2017. Land use data is mainly used for image processing and spatial analysis by means of ENVI 5.1 and ArcGIS 10.2 platforms. The landscape index was calculated using Fragstats 4.2, using 30m×30m grid data unit to calculate the dynamic change pattern of landscape pattern in Guizhou Province from 2000 to 2017. The total value of ecosystem services, the value of individual services and the value flow were measured and analyzed. Finally, SPSS is used to calculate and analyze the impact of landscape pattern changes on the value of ecosystem services. The raster maps of land use classification in Guizhou Province are shown in Figure S1.

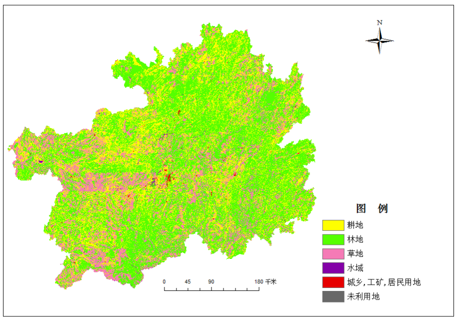

a. Land use classification in 2000

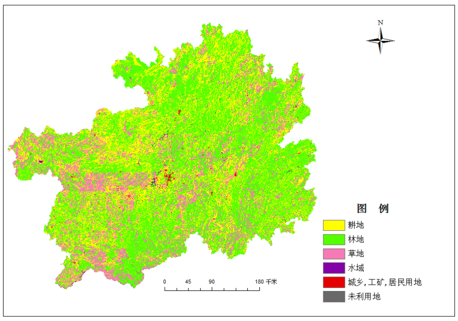

b. Land use classification in

2008

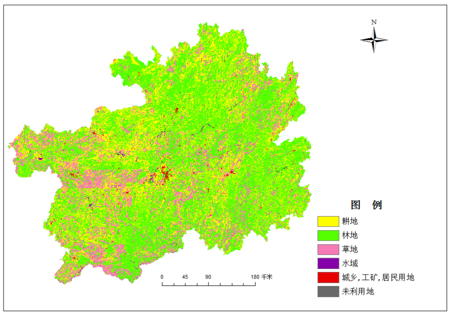

c. Land use classification in 2013

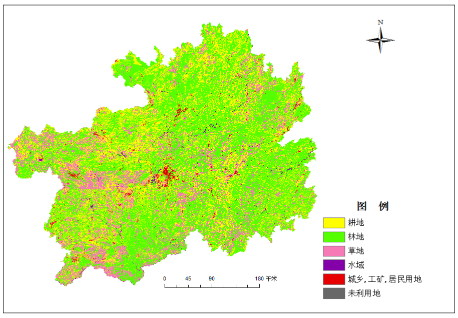

d. Land use classification in 2017

Figure S1 Land use classification of Guizhou Province, China

Based on the Markov transfer matrix model, the spatial superposition analysis tool in ArcGIS was used to superimpose the four remote sensing images of 2000, 2008, 2013 and 2017 to obtain the land use transfer matrix, as shown in Tables S1, S2 and S3.

Table S1 Land use transfer matrix of Guizhou Province from 2000 to 2008（ha）

2000	2008							
	Cropland	Forestland	Grassland	Waters	Urban and rural areas, industrial and mining, residential land	Unutilized land	Total transfer	Transfer ratio %
Cropland	4618053.327	256539.900	82377.014	3291.781	6209.661	38.750	348457.107	7.016
Forestland	219807.600	9056016.655	68846.437	2562.305	3085.644	80.950	294382.936	3.148
Grassland	119069.155	179165.154	2882959.441	4261.159	2490.833	63.579	305049.881	9.568
Waters	908.635	1119.455	506.791	37285.547	42.048	2.373	2579.301	6.449
Urban and rural areas, industrial and mining, residential land	1993.280	942.791	608.514	79.292	56439.003	0.267	3624.144	6.034
Unutilized land	163.810	787.852	75.973	0.646	0.038	3008.423	1028.319	25.473
Total transfer	341942.480	438555.152	152414.729	10195.182	11828.225	185.919	955121.688	
Transfer ratio %	6.894	4.618	5.021	21.410	17.325	5.820		
Net out	6514.626	-144172.216	152635.152	-7615.880	-8204.081	842.400		

Table S2 Land use transfer matrix of Guizhou Province from 2008 to 2013（ha）

2008	2013							
	Cropland	Forestland	Grassland	Waters	Urban and rural areas, industrial and mining, residential land	Unutilized land	Total transfer	Transfer ratio %
Cropland	4628623.506	211560.479	69596.362	9430.681	40662.236	164.634	331414.391	6.681
Forestland	203905.306	9219457.658	44528.602	11037.040	15662.767	97.809	275231.525	2.898
Grassland	73515.863	108894.542	2836568.666	3952.316	12234.512	237.349	198834.582	6.550
Waters	947.172	1268.965	624.653	44553.922	50.218	36.062	2927.070	6.147
Urban and rural areas, industrial and mining, residential land	2498.947	1214.786	852.856	165.063	63538.270	0.038	4731.692	6.931

Unutilized land	38.230	92.096	225.144	8.693	73.160	2757.02	437.323	13.69
Total transfer	280905.520	323030.867	115827.617	24593.793	68682.892	535.892	813576.581	
Transfer ratio %	5.721	3.385	3.923	35.477	51.944	16.273		
Net out	50508.871	-47799.343	83006.965	-21666.723	-63951.201	-98.569		

Table S3 Land use transfer matrix of Guizhou Province from 2013 to 2017（ha）

2013	2017							
	Cropland	Forestland	Grassland	Waters	Urban and rural areas, industrial and mining, residential land	Unutilized land	Total transfer	Transfer ratio %
Cropland	4539519.666	201236.463	68752.902	12922.452	86875.639	141.270	369928.726	7.535
Forestland	206120.172	9246489.38	39409.186	21513.543	28167.573	394.985	295605.459	3.097
Grassland	67426.038	42336.237	2810870.589	8065.108	23412.641	159.407	141399.432	4.789
Waters	1118.153	1792.184	893.162	65013.280	265.306	1.445	4070.250	5.871
Urban and rural areas, industrial and mining, residential land	5010.135	3233.593	2224.953	823.862	120913.558	14.465	11307.009	8.551
Unutilized land	44.429	76.440	160.487	112.993	116.545	2781.936	510.893	15.514
Total transfer	279718.928	248674.917	111440.691	43437.958	138837.704	711.572	822821.769	
Transfer ratio %	5.804	2.619	3.813	39.956	53.449	20.368		
Net out	90209.798	46930.542	29958.741	-39367.708	-127530.695	-200.678		

Table S4 Landscape index equation and ecological meaning

Index	Equation	Ecological meaning
Patch Number（NP）	NP=N<br>At the patch type level, it is equal to the total number of patch types in the landscape; at the landscape level, it is equal to the total number of all patches in the landscape. No unit, range：NP≧1。	Reflecting the spatial pattern of the landscape, the value of the value is positively correlated with the fragmentation degree of the landscape. The larger the NP value, the higher the fragmentation degree; the smaller the NP, the lower the fragmentation degree.
Patch Density（PD）	PD=N/A<br>Where: N is the number of patches in the landscape, and A is the area of a certain type of patch, ranging from PD>1.	The patch density is the number of patches per square kilometer of a patch. It is often used to describe the degree of spatial heterogeneity of landscapes and the degree of fragmentation of landscape patches. The larger the value of PD, the greater the spatial heterogeneity of patches and the higher the degree of fragmentation.

Mean of Patch Area（AREA_MN）	$$\mathrm{AREA\_MN} = \frac{A}{N}$$ Where: A is the total area of the patch of a certain landscape type, N is the number of patches of a certain type of landscape, the unit is ha, and the value range is: AREA_MN>0。	Reflecting the average patch size of a landscape type and the degree of fragmentation of landscape types. It can also describe the differences between different landscape types. The smaller the value, the greater the degree of landscape fragmentation; the larger the value, the smaller the landscape fragmentation.
Largest Patch Index（LPI）	$$LPI = \frac{\max_{j=1}^{n}(a_{ij})}{A}(100)$$ Where: a_{ij} indicates the area of the patch ij; A is the total area of the landscape including the interior background of the landscape. LPI is the division of the largest patch area in a patch type by the entire landscape area, then multiplied by 100 to convert to a percentage. The range of values is 0<LPI<100。	When it is close to 0, the smaller the area of the largest patch in this type of patch is. When it is equal to 100, the entire landscape consists of a patch; it helps to determine the type of advantage of the landscape.
Landscape Shape Index（LSI）	$$LSI = \frac{E}{\min E}$$ Where: The E value is the total length of the edge of the landscape, including all landscape boundaries and background edges. Min E is the smallest possible value of E.	When LSI is equal to 1, it means that there is only one patch in the landscape. With the irregular shape of the landscape and the lengthening of the edge, the LSI becomes larger and has no maximum limit.
Area-weighted Mean Shape Index（SHAPE_AM）	$$\mathrm{SHAPE\_AM} = \sum_{j=1}^{n}\left[X_{ij}\left(\frac{a_{ij}}{\sum_{j=1}^{n} a_{ij}}\right)\right]$$ Where: n is the number of patches of a certain type in the landscape, X_{ij} is the perimeter of the patch ij, a_{ij} is the area of the patch ij, and the range of values: SHAPE_AM≧1。	It is one of the important indicators to measure the complexity of the landscape spatial pattern. When the value is equal to 1, the shape of all patches is the simplest square; the larger the value, the more complicated and irregular the surface patch shape; the smaller the value, the simpler and more regular the patch shape.
Aggregation Index（AI）	$$AI = \left[\sum_{i=1}^{m}\left(\frac{g_{ij}}{\max \longrightarrow g_{ij}}\right)p_i\right](100)$$ Where: g_{ij} is the critical amount between patches of landscape type ij, $\max \longrightarrow g_{ij}$ is the maximum critical value between patches of landscape type ij.	The degree of convergence reflects the degree of interconnection between the same types of landscape patches. When the degree of fragmentation of a land type is the most clustered, the AI value approaches 0; when the aggregation degree of a land type is the strongest, the AI value Approaching 100.
Perimeter-Area Fractal Dimension（PAFRAC）	$PAFRAC =$ Wher $$\frac{2}{\frac{\left[N\sum_{i=1}^{m}\sum_{j=1}^{n}(\ln p_{ij}\cdot \ln a_{ij})\right]-\left[(\sum_{i=1}^{m}\sum_{j=1}^{n}\ln p_{ij})(\sum_{i=1}^{m}\sum_{j=1}^{n}\ln a_{ij})\right]}{(N\sum_{i=1}^{m}\sum_{j=1}^{n}\ln p_{ij}^{2})-(\sum_{i=1}^{m}\sum_{j=1}^{n}\ln p_{ij})}}$$	The size of the PAFRAC value reflects the complexity of the shape of the landscape patch. When the value is closer to 2, the shape of the plaque is more complicated and irregular, and the

	e: a_{ij} is the area of a plaque, N is the total number of patches, p_{ij} is the perimeter of a patch, and the range of values 1≦PAFRAC≦2。	degree of influence of human activities on the landscape is smaller. When the value is closer to the value At 1 o'clock, the simpler and more regular the patch shape, the higher the self-similarity of the patch and the greater the degree of influence by human activities.
Contagion Index（CONTAG）	$CONTAG = \left\{ 1 + \frac{\sum_{i=1}^{m}\sum_{k=1}^{m}\left[(p_i)(\frac{g_{ii}}{\sum_{k=1}^{m} g_{jk}})\left[\ln(p_i)(\frac{g_{ik}}{\sum_{k=1}^{m} g_{jk}})\right]\right]}{2\ln(m)} \right\}$ Where: the area specific gravity of the p-value patch type i in the landscape; g is the number of nodes between the patch type i and the patch type k based on the double method; m is the number of patch types in the landscape, including the landscape boundary The type of patch in the indicator; the unit of the indicator is %, and the range of values is: 0<CONTAG《100.	When all patch types are maximally fragmented and intermittently distributed, the indicator approaches 0; when the patch types are maximally concentrated together, the indicator reaches 100. The index contains spatial information about the landscape, mainly describing the different types of aggregation and extension of the landscape.
Interspersion and Juxtaposition index（IJI）	$IJI = -\sum_{k=1}^{m}\left[(\frac{e_{ik}}{\sum_{k=4}^{m} e_{ik}})\ln(\frac{e_{ik}}{\sum_{k=4}^{m} e_{ik}})\right] \times \frac{100}{\ln(m-1)}$ Where : m is the total number of patch types, e_{ik} is the total edge length between patch type i and patch type k, in the range of 0<IJI≦100.	Reflecting the overall Interspersion and Juxtaposition between landscape types, indicating the relationship between landscape patch types, when the value is closer to 0, it indicates that the less patch of a certain type is connected with other types of patches; When the value approaches 100, it indicates that the more patch of a certain type is connected with other types of patch; when the value is 100, it indicates that the connection between the patch and other surrounding patches is equal, there are Typical artificial landscape features.
Shannon Diversity Index（SHDI）	$SHDI = -\sum_{i=1}^{N}\left[p_i \ln(p_i)\right]$ Where: p_i is the area specific gravity of the patch type i in the landscape. SHDI is equal to the sum of the area ratio of each patch type in the landscape and the logarithm of the natural number, and then the opposite. The indicator has a value range of SHDI≧0。	When there is only one patch in the entire landscape, SHDI=0. With the increase of the number of patch types in the landscape and the equalization of their area proportions, the SHDI value increases; the index is widely used in ecology and is sensitive to the unbalanced distribution of patch types in the landscape. The richer the patch type, the higher the degree of fragmentation; the greater the amount of uncertain information, the higher the value of SHDI obtained.
Shannon Evenness Index（SHEI）	$SHEI = \frac{-\sum_{i=1}^{n}\left[p_i \ln(p_i)\right]}{\ln(m)}$	As the proportion of different patch types in the landscape becomes more and more unbalanced,

	Where p_i is the area specific gravity of the patch type i in the landscape, the landscape area used in the calculation does not include the background in the landscape; m refers to the number of patch types in the landscape; SHEI is equal to the Shannon's diversity index and the number of plaque types. Logarithmic ratio; the index has no unit; the index has a value range of 0≦SHEI≦1	the index value keeps approaching 0; when there is only one patch in the whole landscape, SHEI=0; when the patch type area proportion in the landscape is the same, SHEI =1.

Table S5 Indices of landscape pattern at land cover type level

Num	Index
1	NP
2	PD
3	AREA_MN
4	LSI
5	SHAPE_AM
6	AI
7	IJI

Table S6 Indices of landscape pattern at Landscape level

Num	Index
1	NP
2	PD
3	LSI
4	LPI
5	PAFRAC
6	IJI
7	CONTAG
8	SHDI
9	SHEI

Table S7 Values of landscape pattern indices on land cover type level (Unit: 10^{10} Yuan)

Year	Land use type	NP	PD	LSI	AREA_MN	SHAPE_AM	IJI	AI
2000	Cropland	73452	0.417	621.739	67.619	19.328	39.693	91.643
	Forestland	19633	0.112	402.983	476.313	200.642	32.004	96.056
	Grassland	17142	0.097	327.775	185.999	13.090	44.511	94.509
	Waters	1352	0.008	69.624	29.584	6.597	69.055	89.685
	Urban and rural areas, industrial and mining, residential land	2417	0.014	71.133	24.851	3.035	63.783	91.404
	Unutilized land	134	0.001	16.955	30.127	2.100	67.206	92.423
2008	Cropland	68633	0.390	624.104	72.344	19.711	39.278	91.609

	Forestland	18225	0.104	403.044	520.808	199.196	31.359	96.085
	Grassland	14145	0.080	322.662	214.544	13.702	44.831	94.460
	Waters	943	0.005	71.419	50.513	6.922	69.649	90.301
	Urban and rural areas, industrial and mining, residential land	2513	0.014	73.038	27.243	3.142	63.248	91.728
	Unutilized land	111	0.001	15.987	28.843	2.187	69.147	91.995
2013	Cropland	71488	0.406	610.797	68.680	17.796	40.216	91.742
	Forestland	19797	0.112	394.289	482.070	197.462	33.055	96.180
	Grassland	16094	0.091	315.820	183.468	13.265	46.934	94.503
	Waters	1308	0.007	73.199	53.000	7.382	69.341	91.761
	Urban and rural areas, industrial and mining, residential land	4020	0.023	89.535	32.892	2.930	66.428	92.687
	Unutilized land	124	0.001	16.201	26.557	2.114	72.157	92.002
2017	Cropland	73054	0.415	624.792	65.973	16.550	43.511	91.474
	Forestland	19664	0.112	401.877	482.945	181.855	36.957	96.097
	Grassland	15600	0.089	320.510	187.355	12.800	50.726	94.392
	Waters	1194	0.007	111.086	91.050	9.807	69.637	89.970
	Urban and rural areas, industrial and mining, residential land	7830	0.045	130.391	33.175	4.526	68.274	92.379
	Unutilized land	171	0.001	18.709	20.431	2.090	73.449	90.944

Table S8 Values of landscape pattern indices on landscape level (Unit: 10^{10} Yuan)

Year	NP	PD	LPI	LSI	PAFRAC	CONTAG	IJI	SHDI	SHEI
2000	114130	0.648	36.229	387.133	1.470	64.269	37.292	1.038	0.579
2008	104570	0.594	36.730	386.436	1.586	64.441	36.996	1.032	0.576
2013	112831	0.641	37.266	378.908	1.466	64.076	38.686	1.048	0.585
2017	117513	0.667	34.689	390.257	1.560	62.895	42.921	1.081	0.603

Table S9 values of ecosystem services (Unit: 10^{10} Yuan)

Item	2000	percentage %	2008	Percentage %	2013	Percentage %	2017	Percentage %
Gas regulation	3.787	14.712	3.842	14.745	3.858	14.738	3.837	14.691
Climate regulation	3.096	12.028	3.137	12.038	3.147	12.024	3.129	11.981
Water conservation	3.58	13.907	3.64	13.959	3.675	14.040	3.7	14.165
Waste treatment	2.238	8.694	2.247	8.626	2.262	8.640	2.283	8.740
Soil formation and protection	4.554	17.691	4.61	17.695	4.624	17.663	4.595	17.593

Biodiversity conservation	3.764	14.620	3.807	14.613	3.821	14.595	3.804	14.563
Food production	0.643	2.498	0.632	2.424	0.623	2.379	0.615	2.354
Raw material	2.718	10.559	2.76	10.592	2.773	10.594	2.759	10.564
Entertainment culture	1.362	5.291	1.383	5.309	1.395	5.327	1.397	5.349
Total	25.743		26.058		26.178		26.119	
